# Ceramide targets xIAP and cIAP1 to sensitize metastatic colon and breast cancer cells to apoptosis induction to suppress tumor progression

**DOI:** 10.1186/1471-2407-14-24

**Published:** 2014-01-15

**Authors:** Amy V Paschall, Mary A Zimmerman, Christina M Torres, Dafeng Yang, May R Chen, Xia Li, Erhard Bieberich, Aiping Bai, Jacek Bielawski, Alicja Bielawska, Kebin Liu

**Affiliations:** 1 Department of Biochemistry and Molecular Biology, Medical College of Georgia, Georgia Regents University, Augusta, GA, USA; 2 Institute of Molecular Medicine and Genetics, Medical College of Georgia, Georgia Regents University, Augusta, GA, USA; 3 Department of Biochemistry and Molecular Biology, Medical University of South Carolina, Charleston, SC 29425, USA; 4 Cancer Center, Georgia Regents University, Augusta, GA 30912, USA

**Keywords:** Ceramide, xIAP, cIAP1, Bcl-xL, Fas, Apoptosis sensitization

## Abstract

**Background:**

Ceramide is a bioeffector that mediates various cellular processes, including apoptosis. However, the mechanism underlying ceramide function in apoptosis is apparently cell type-dependent and is not well-understood. We aimed at identifying molecular targets of ceramide in metastatic human colon and breast cancer cells, and determining the efficacy of ceramide analog in suppression of colon and breast cancer metastasis.

**Methods:**

The activity of and mechanism underlying ceramide as a cytotoxic agent, and as a sensitizer for Fas-mediated apoptosis was analyzed in human cell lines established from primary or metastatic colon and breast cancers. The efficacy of ceramide analog LCL85 in suppression of metastasis was examined in preclinical mouse tumor models.

**Results:**

Exposure of human colon carcinoma cells to ceramide analog LCL85 results in apoptosis in a dose-dependent manner. Interestingly, a sublethal dose of LCL85 increased C16 ceramide content and overcame tumor cell resistance to Fas-mediated apoptosis. Subsequently, treatment of tumor cells with exogenous C16 ceramide resulted in increased tumor cell sensitivity to Fas-mediated apoptosis. LCL85 resembles Smac mimetic BV6 in sensitization of colon carcinoma cells to Fas-mediated apoptosis by inducing proteasomal degradation of cIAP1 and xIAP proteins. LCL85 also decreased xIAP1 and cIAP1 protein levels and sensitized metastatic human breast cancer cells to Fas-mediated apoptosis. Silencing xIAP and cIAP1 with specific siRNAs significantly increased the metastatic human colon carcinoma cell sensitivity to Fas-mediated apoptosis, suggesting that IAP proteins mediate apoptosis resistance in metastatic human colon carcinoma cells and ceramide induces IAP protein degradation to sensitize the tumor cells to apoptosis induction. Consistent with its apoptosis sensitization activity, subtoxic doses of LCL85 suppressed colon carcinoma cell metastatic potential in an experimental lung metastasis mouse model, as well as breast cancer growth and spontaneous lung metastasis in an orthotopic breast cancer mouse model.

**Conclusion:**

We have identified xIAP and cIAP1 as molecular targets of ceramide and determined that ceramide analog LCL85 is an effective sensitizer in overcoming resistance of human cell lines established from metastatic colon and breast cancers to apoptosis induction to suppress metastasis *in vivo*.

## Background

Fas (also termed CD95 and TNFRSF6) is a member of the TNF death receptor superfamily. Despite other “non-apoptotic” cellular responses emanating from its signaling, the major and best known function of Fas is apoptosis. Fas is expressed on tumor cell surface, and its physiological ligand, FasL, is expressed on activated T cells and NK cells. Compelling experimental data from both human cancer patients and mouse tumor models indicate that the Fas-mediated apoptosis pathway plays a key role in suppression of cancer development and in host cancer immunosurveillance [1-3]. Furthermore, human cancer genomics data indicate that Fas is not significantly focally amplified across a dataset of 3131 tumors, but is significantly focally deleted across the entire dataset of these 3131 tumors, including human colorectal cancer (http://www.broadinstitute.org/tumorscape/pages/portalHome.jsf). These data thus strongly suggest that Fas functions as a tumor suppressor.

To avoid apoptosis, tumor cells tend to down-regulate Fas expression or alter the expression of key mediators of the Fas-mediated apoptosis signaling pathway to advance the disease [4-6]. This is well-supported by the phenomenon that resistance to apoptosis, including Fas-mediated apoptosis, is a hallmark in human cancers [5,7], particularly in metastatic human colorectal cancer [2,3,8] and breast cancer [9]. Therefore, therapeutic intervention of tumor cell resistance to Fas-mediated apoptosis potentially represents an effective approach to render tumor cell sensitivity to FasL^+^ cytotoxic T lymphocytes (CTL) of the host immunosurveillance system or to CTL-based adoptive cancer immunotherapy to suppress tumor progression [1,5,10].

During the last decade, sphingolipids have emerged as bioeffectors that mediate various cellular processes, including proliferation and apoptosis of cancer cells [11-14]. Sphingolipid deregulation, namely the balance between ceramide and sphingosine 1-phosphate, has been implied as a key factor in tumor pathogenesis and apoptosis resistance [13,15]. Although it has been demonstrated that de novo-generated ceramides may confer certain types of tumor cells with resistance to apoptosis [16], ceramide, the central molecule of the sphingolipid metabolism pathway, generally promotes apoptosis [17-19]. The role of ceramide in Fas-mediated apoptosis has also been well-documented [20]. Ceramide enables Fas receptor to cluster to increase Fas-mediated apoptosis [21], and modulate Fas receptor activation [22,23]. Ceramide has also been shown to regulate apoptosis through modulating key molecules of the Fas-mediated apoptosis pathways [22,24-26]. Elevation of acid ceramidase, the enzyme that converts ceramide to sphingosine and subsequently sphingosine 1-phosphate, has been frequently observed in apoptosis-resistant cancer cells, including metastatic colon carcinoma cells [17,27,28]. These observations thus suggest that targeting ceramide metabolism to increase ceramide accumulation might be an effective approach to overcome cancer cell resistance to Fas-mediated apoptosis. In this study, we demonstrated that aromatic ceramide analog LCL85 effectively overcomes metastatic human colon and breast cancer cell resistance to Fas-mediated apoptosis at least partially through inducing proteasomal degradation of cIAP1 and xIAP *in vitro*. More significantly, we demonstrated that LCL85 effectively suppresses colon and breast cancer metastasis *in vivo*. Our data determined that LCL85 is potentially an effective apoptosis sensitizer that warrants further development as an adjunct agent to increase the efficacy of FasL^+^ CTL-based cancer immunotherapy.

## Methods

### Mice

BALB/c mice were obtained from National Cancer Institute (Frederick, MD). All studies are approved by the Georgia Regents University Institutional Animal Care and Use Committee (Protocol# 2011–0365).

### Cell lines

All human cell lines established from primary and metastatic colon and breast cancer tissues (referred to as primary and metastatic human colon and breast cancer cell lines), and mouse breast cancer cell line 4 T1 were obtained from American Type Culture Collection (ATCC) (Manassas, VA). ATCC characterizes these cells by morphology, immunology, DNA fingerprint, and cytogenetics. Murine Colon26 cells were kindly provided by Dr. William E. Carson, III (Ohio State University, Columbus, OH).

### Reagents

BV6 was kindly provided by Genentech. Ceramide analogs B13 and LCL85 were synthesized by Lipidomics Shared Resource at Medical University of South Carolina [29]. FasL (Mega-Fas Ligand®) was provided by Drs. Steven Butcher and Lars Damstrup (Topotarget A/S, Denmark). C16-ceramide was obtained from Santa Cruz Biotech, and was dissolved in dodecane:ethanol (2:98, v/v; 0.05% final concentration) as described [21]. MG-132 and Z-VAD-FMK were obtained from Enzo Life Sciences (Farmingdale, NY).

### Western blotting analysis

Western blotting analysis was performed as previously described [30]. Anti-cIAP1 was obtained from R&D System (Minneapolis, MN). Anti-Bax and cIAP2 antibodies were obtained from Santa Cruz Biotech (Santa Cruz, CA). Anti-Bak and xIAP antibodies were obtained from Cell Signaling Biotech (Danvers, MA), anti-Bcl-2, and Bcl-xL antibodies were obtained from BD Biosciences (San Diego, CA), and anti-β-actin was obtained from Sigma (St Louis, MO).

### Cell viability assays

Cell viability assay was carried out as previously described [31] using the MTT cell proliferation assay kit (ATCC, Manassas, VA).

### Apoptosis analysis

Cells were treated with BV6, LCL85, or C16 ceramide for 1 h, followed by incubation with FasL for approximately 24 h. Apoptosis analysis was as previously described [32]. Briefly, cells were then collected and incubated with propidium iodide (PI) and Annexin V (Biolegend), and analyzed by flow cytometry. The percentage of apoptosis was calculated by the formula: % apoptosis = % PI and AnnexinV double positive cells with FasL - % PI and Annexin V double positive cells without FasL.

### Measurement of endogenous ceramide level

Cellular levels of endogenous ceramides were measured by Lipidomics Shared Resource, MUSC, using high-performance liquid chromatography-mass spectrometry approach (LC-MS/MS) as previously described. Ceramide levels were normalized to the total cellular protein contents.

### Cell surface protein analysis

Tumor cells were stained with anti-Fas (BD biosciences), anti-FasL (BD biosciences), or anti-CD8 (Biolegend, San Diego, CA) mAbs. Isotype-matched control IgG (Biolegend) was used as a negative control. The stained cells were analyzed by flow cytometry. For FasL protein analysis, mouse lungs were digested in collagenase solution to make a single cell suspension. The cell suspension was stained with PE-conjugated FasL (BD Biosciences) or FITC-conjugated CD8 mAb, or both mAbs and analyzed by flow cytometry.

### Gene silencing

RNAi-based silencing of gene expression in tumor cells was done as previously described [33]. Briefly, SW620 cells were transiently transfected with scramble siRNA (Dharmacon), and human xIAP- and cIAP1-specific siRNAs (Santa Cruz Biotech), respectively, using Lipofectamine 2000 (Invitrogen) for approximately 24 h. Cells were then harvested. Part of the cells were used for RT-PCR analysis of xIAP and cIAP expression. Another part of the cells were cultured in the absence or presence of FasL for approximately 24 h and then analyzed for apoptosis.

### Liver toxicity analysis

LCL85 was injected to BALB/c mice (5 mg/kg body weight) i.v. Peripheral blood was collected from mice 3 days later using Multivette 600 Z-gel tubes (SARSTEDT). Serum was separated by centrifugation and measured for complete liver enzyme profile at Georgia Laboratory Animal Diagnostic Service (Athens, GA).

### Colon cancer experimental lung metastasis

Colon 26 cells (1.5×10^5^ cells/mouse) were injected to BALB/c mice iv. LCL85 (0, 1 and 5 mg/kg body weight) was injected iv to tumor-bearing mice at days 3, 6, 9 and 12 after tumor injection. Mice were sacrificed at day 14 and analyzed for lung metastasis as previously described [34].

### Breast cancer spontaneous lung metastasis

4 T1 cells (1×10^4^ cells/mouse) were injected to the mammary fat pad. LCL85 (2.5 mg/kg body weight) was injected to the tumor-bearing mice at days 7, 10, 13, and 16 after tumor injection. Mice were sacrificed 29 days after tumor injection, and analyzed for primary tumor growth and lung metastasis. To determine the efficacy of LCL85 on metastasis, 4 T1 cells (1×10^4^ cells/mouse) were injected to the mammary fat pad. Primary tumors were surgically removed 16 days later. Mice were treated with LCL85 (2.5 mg/kg body weight) at days 10, 13, and 16 after surgery. Mice were sacrificed and analyzed for lung metastasis 19 days after surgery.

### Statistical analysis

Where indicated, data were represented as the mean ± SD. Statistical analysis was performed using two-sided *t* test, with *p*-values < 0.05 considered statistically significant.

## Results

### Ceramide analog effectively sensitizes metastatic human colon and breast cancer cell apoptosis resistance

**Figure 1:**
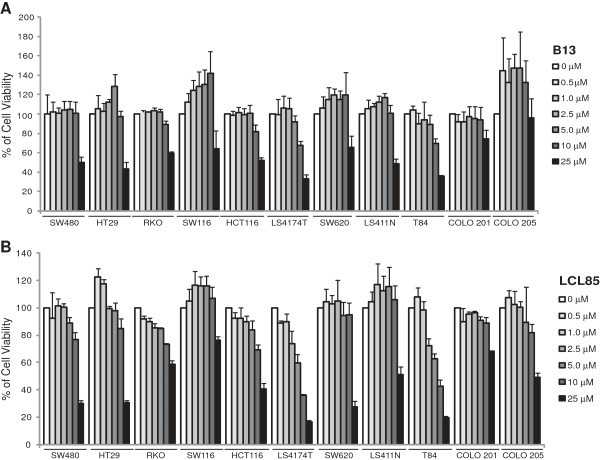
**Cytotoxicity of ceramide analogs to human colon carcinoma cells. A & B.** Eleven human colon carcinoma cell lines were cultured with B13 **(A)** and LCL85 **(B)** at the indicated doses for 24 h, respectively. Cell growth rates were measured by MTT assays. The cell growth rates in the control groups (without ceramide analogs) were arbitrarily set as 100%. Column, mean; bar, SD.

**Figure 2:**
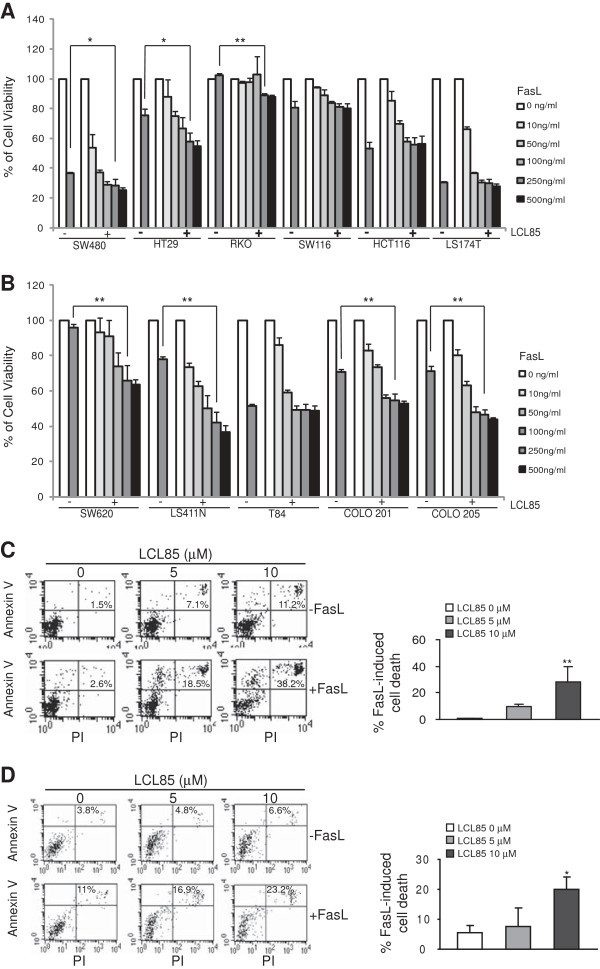
**A suboptimal dose of ceramide analog LCL85 overcomes metastatic human colon carcinoma cell resistance to Fas-mediated apoptosis. A & B**. Sensitization of primary **(A)** and metastatic **(B)** human colon carcinoma cells to FasL-induced growth inhibition by LCL85. Six primary human colon carcinoma cell lines **(A)** and 5 metastatic human colon carcinoma cell lines **(B)** were cultured in the absence or presence of a sublethal dose of LCL85 (5 μM) and FasL at the indicated concentrations for 24 h. Cell growth rate was measured by MTT assays. *p < 0.05; **p < 0.01. **C & D**. SW620 **(C)** and LS411N **(D)** cells were incubated with LCL85 at the indicated dose for 1 h and then cultured in the absence or presence of FasL (250 ng/ml) overnight. Cells were stained with Annexin V and PI and analyzed by flow cytometry. Cell death is indicated at the top right corner of each plot. Percentage of FasL-induced cell death is calculated by the formula: % Annexin V^+^ and PI^+^ cells in the presence of FasL- % Annexin V^+^ and PI^+^ cells in the absence of FasL, and presented at the right.

Ceramide analogs of B13 and LCL85 were tested for their cytotoxicity against human colon carcinoma cell lines (6 primary and 5 metastatic human colon cancer cell lines). Cell growth inhibition assays indicated that B13 and LCL85 are both cytotoxic at high doses (Figure 1). LCL85 represents a unique compound since it is highly cytotoxic at high doses, but exhibited almost no cytotoxicity at low doses (Figure 1B). Because our objective was to test the hypothesis that ceramide analogs are effective apoptosis sensitizers for Fas-mediated apoptosis in human colon carcinoma cells, we chose LCL85 for this study. Next, eleven human colon carcinoma cell lines were cultured in the presence of a sublethal dose of LCL85 (5 μM) and various doses of FasL, and analyzed for tumor cell viability. Four of the 6 primary colon carcinoma cell lines (SW480, HT29, HCT116 and LS174T) are highly sensitive to FasL-induced apoptosis, and LCL85 exhibited minimal or no sensitization effects on these 4 sensitive cell lines (Figure 2A). On the other hand, the other 2 primary human colon carcinoma cell lines RKO and SW116 are resistant to Fas-mediated apoptosis. However, LCL85 also only exhibited minimal or no sensitization effects on these 2 cell lines (Figure 2A). One of the 5 metastatic human colon carcinoma cell lines (T84) is sensitive to FasL-induced apoptosis, but 4 of the 5 metastatic human colon carcinoma cell lines (SW620, LS174T, Colo201 and Colo205) are resistant to Fas-mediated apoptosis. A sublethal dose of LCL85 significantly increased these 4 metastatic human colon carcinoma cell lines to FasL-induced apoptosis (Figure 2B). In summary, our data demonstrated that a sublethal dose of LCL85 is effective in sensitizing the apoptosis-resistant human colon carcinoma cells to Fas-mediated apoptosis.

Next, we used SW620 and LS411N cells to determine whether the above observed tumor cell growth inhibition is due to apoptosis. SW620 and LS411N cells were cultured in the presence of LCL85 and FasL, and analyzed for apoptosis. Staining cells with Annexin V and PI revealed that LCL85 induces apoptosis of SW620 and LS411N cells in a dose-dependent manner. However, LCL85 alone at low doses only induced a small degree of apoptosis (Figure 2C). In contrast, a sublethal dose of LCL85 dramatically increased SW620 and LS411N cell sensitivity to FasL-induced apoptosis (Figure 2C & D).

**Figure 3:**
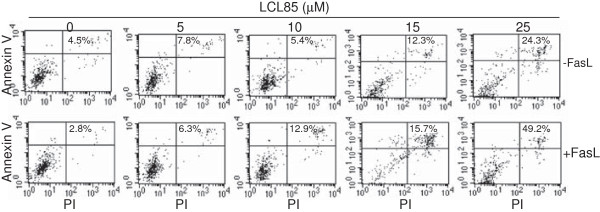
**LCL85 overcomes metastatic breast cancer cell resistance to Fas-mediated apoptosis.** MDA-MB-231 cells were incubated with LCL85 at the indicated doses in the absence or presence of FasL (250 ng/ml) for approximately 24 h. Cells were stained with Annexin V and PI and analyzed by flow cytometry. Cell death was indicated at the top right corner of each plot.

To determine whether LCL85-sensitized apoptosis is tumor type-dependent, we also tested the effects of LCL85 on metastatic human breast cancer cells. MDA-MB-231 cells were treated with various doses of LCL85 in the absence or presence of FasL and analyzed for apoptosis. As in the human colon carcinoma cells, LCL85 induced MDA-MB-231 apoptosis in a dose-dependent manner, albeit at a low degree (Figure 3). MDA-MB-231 cells are resistant to FasL-induced apoptosis, and LCL85 is effective in sensitizing MDA-MB-231 cells to FasL-induced apoptosis at a dose of 25 μM (Figure 3). These observations thus suggest that a sublethal dose of ceramide analog LCL85 is a potent apoptosis sensitizer.

### LCL85 increases cellular C16 ceramide level to sensitize colon carcinoma cells to apoptosis

**Figure 4:**
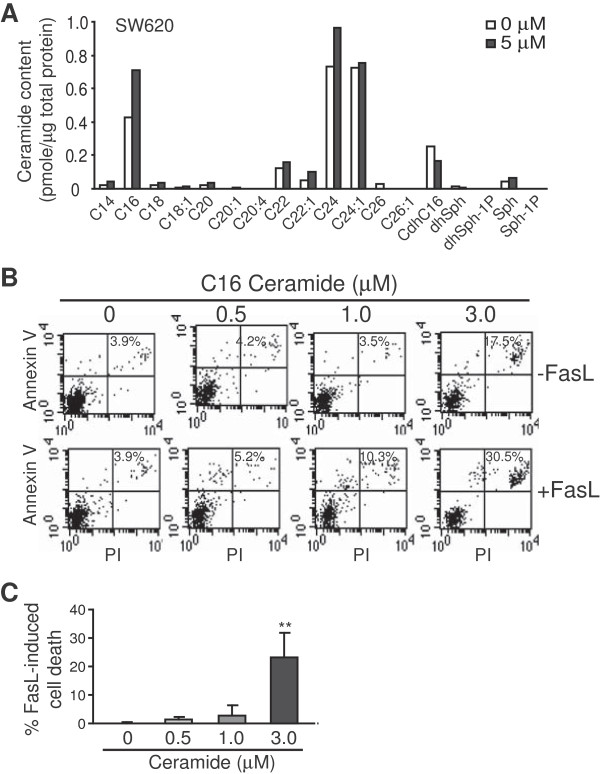
**C16 ceramide sensitizes metastatic human colon carcinoma cells to Fas-mediated apoptosis. A**. SW620 cells were treated with LCL85 (5 μM) for 24 h. Cells were then lysed and measured for ceramide contents. Shown are representative results of one of two independent measurements. **B**. Exogenous C16 ceramide sensitizes tumor cell to Fas-mediated apoptosis. SW620 cells were treated with C16 ceramide for 1 h at the indicated concentrations, and then cultured in the absence or presence of FasL for approximately 24 h. Cells were stained with Annexin V and PI and analyzed by flow cytometry. The apoptotic cell death is indicated at the top right corner of each plot. Shown are results of one of three representative experiments. **C**. Quantification of apoptosis as shown in B. % FasL-induced cell death is calculated by the formula: % Annexin V^+^ and PI^+^ cells in the presence of FasL- % Annexin V^+^ and PI^+^ cells in the absence of FasL. Column: mean; Bar: SD.

We next treated SW620 cells with a sublethal dose of LCL85 and measured the level of cellular ceramides and ceramide metabolites. Treatment of LCL85 increased C16 ceramide level in the tumor cells (Figure 4A), suggesting that LCL85 might increase C16 ceramide level to sensitize human colon carcinoma cells to Fas-mediated apoptosis. To test this hypothesis, SW620 cells were cultured in the presence of exogenous C16 ceramide and FasL. Although exogenous C16 ceramide directly induced apoptosis in a dose-dependent manner, albeit at a low level, exogenous C16 ceramide significantly increased SW620 cell sensitivity to FasL-induced apoptosis (Figure 4B & C). Therefore, LCL85 sensitizes human colon carcinoma cells to Fas-mediated apoptosis at least partially through increasing C16 ceramide level in the tumor cells.

### xIAP and cIAP1 are molecular targets of LCL85

**Figure 5:**
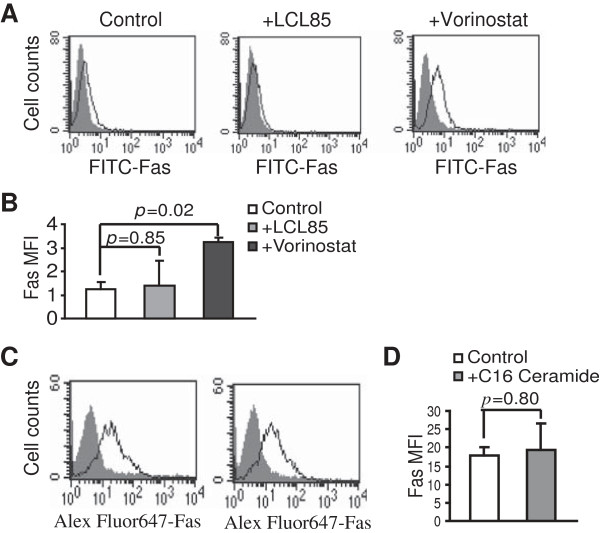
**Ceramide does not alter Fas receptor expression. A**. Cell surface Fas protein level. SW620 cells were cultured in the presence of LCL85 (5 μM) and Vorinostat (0.5 μM) for approximately 24 h and analyzed for cell surface Fas protein level by flow cytometry. Gray area: IgG isotype control staining, solid line: Fas-specific mAb staining. **B**. Quantification of Fas protein level. Cells as shown in A were quantified for Fas protein level by mean fluorescence intensity (MFI). Column, mean; bar, SD. **C**. SW620 cells were cultured in the presence of C16 ceramide (2 μM) for approximately 24 h and analyzed for cell surface Fas protein level by flow cytometry. Gray area: IgG isotype control staining, solid line: Fas-specific mAb staining. **D**. Quantification of FAS MFI as shown in C. Column: mean; bar: SD.

We next sought to identify the targets of ceramide. To determine whether LCL85 alters Fas expression, we treated SW620 cells with LCL85 and analyzed cell surface Fas protein levels. Flow cytometry analysis indicated that LCL85 does not increase cell surface Fas protein level (Figure 5A). As a positive control, Vorinostat significantly increased cell surface Fas protein level in SW620 cells [34] (Figure 5A & B). As a complimentary approach, SW620 cells were treated with C16 ceramide and analyzed for cell surface Fas expression level. C16 ceramide treatment did not alter cell surface Fas protein level (Figure 5C & D).

**Figure 6:**
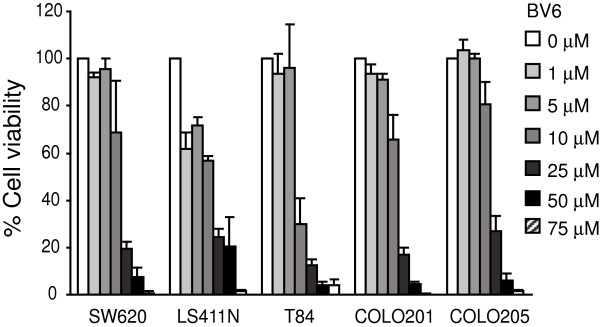
**Cytotoxicity of Smac mimetic to metastatic human colon carcinoma cells.** Five metastatic human colon carcinoma cell lines were cultured in the presence of various doses of BV6 for 24 h. Cell growth rates were measured by MTT assays. The cell growth rates in the control groups (without ceramide analogs) were arbitrarily set as 100%. Column, mean; bar, SD.

**Figure 7:**
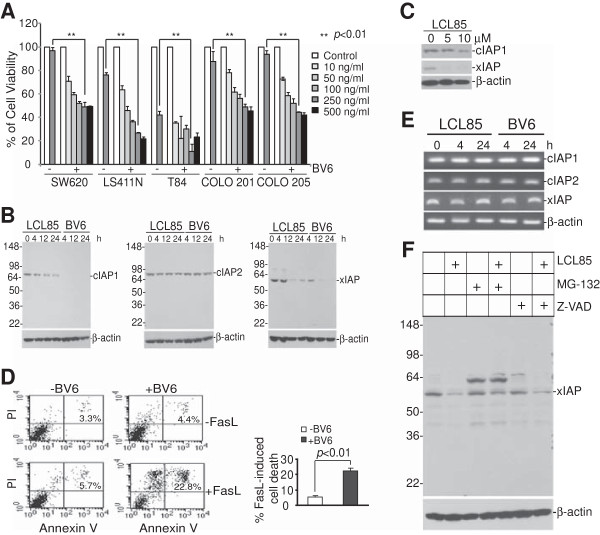
**Ceramide targets cIAP1 and xIAP to sensitize metastatic human colon and breast cancer cells to Fas-mediated apoptosis. A**. Five metastatic human colon carcinoma cell lines were cultured in the absence or presence of BV6 (5 μM) and FasL at the indicated concentrations for 24 h. Cell growth rates were measured by MTT assays. *p < 0.05; **p < 0.01. **B**. SW620 cells were treated with LCL85 (5 μM) for approximately 24 h and analyzed for IAP protein levels at the indicated time points. **C**. MDA-MD-231 cells treated with LCL85 at the indicated doses for approximately 24 h and analyzed for xIAP and cIAP1 protein levels by Western blotting. **D**. SW620 cells were incubated with BV6 (5 μM) for 1 h and then cultured in the absence or presence of FasL overnight. Cells were stained with Annexin V and PI and analyzed by flow cytometry. The apoptotic cell death was indicated at the top right corner of each plot. Right panel: Quantification of apoptosis. % FasL-induced cell death is calculated by the formula: % Annexin V^+^ and PI^+^ cells in the presence of FasL - % Annexin V^+^ and PI^+^ cells in the absence of FasL. **E**. SW620 cells were treated with LCL85 (5 μM) and BV6 (5 μM), respectively, for the indicated time and analyzed for expression levels of cIAP1, cIAP2 and xIAP by RT-PCR. **F**. SW620 cells were pretreated with or without MG-132 (20 μM) or Z-VAD (25 μM) for 1 h followed by treatment with LCL85 for 12 h. Cells were lysed and analyzed for xIAP protein level by Western blotting.

The above observations that LCL85 does not alter Fas level suggests that LCL85 may target mediators of the Fas-mediated apoptosis signaling pathways. IAPs are potent inhibitors of apoptosis, including Fas-mediated apoptosis [35-37]. To determine whether IAPs play a role in metastatic human colon carcinoma apoptosis resistance, we tested the effects of IAP-specific inhibitor BV6 on metastatic human colon carcinoma cells. The same panel of 5 metastatic human colon carcinoma cell lines (Figure 2B) were cultured in the presence of various doses of BV6 and measured for growth inhibition. Like LCL85, BV6 exhibited direct cytotoxicity in a dose-dependent manner (Figure 6). Next, we used a sublethal dose of BV6 to determine whether BV6 sensitizes metastatic human colon carcinoma cells to FasL-induced apoptosis. Incubation of tumor cells with BV6 and FasL revealed that BV6 significantly increases sensitivity of all 5 metastatic human colon carcinoma cells to FasL-induced cell growth inhibition (Figure 7A), and the growth inhibition pattern is strikingly similar to that induced by LCL85 and FasL (Figure 1B), suggesting that LCL85 might sensitize metastatic colon carcinoma cells to Fas-mediated apoptosis by a mechanism similar to BV6.

### BV6 targets IAP proteins to induce apoptosis

We then analyzed the effects of LCL85 on IAP proteins in metastatic human colon carcinoma cells. SW620 cells were treated with LCL85 and analyzed for IAP protein levels at various time points. Among the 3 IAP proteins, xIAP protein levels dramatically decreased 12 h after LCL85 treatment. cIAP1 protein was also decreased, albeit at a smaller degree. cIAP2 protein level was not significantly changed by LCL85 treatment (Figure 7B). To determine whether LCL85 also decreases xIAP protein levels in metastatic human breast cancer cells, MDA-MB-231 cells were treated with LCL85, and analyzed for xIAP and cIAP protein levels. It is clear that LCL85 decreases xIAP and cIAP1 protein levels in a dose-dependent manner (Figure 7C). Next, SW620 cells were cultured in the presence of a sublethal dose of BV6 (5 μM) and FasL, and analyzed for apoptosis. It is clear that BV6 dramatically increased SW620 cell sensitivity to FasL-induced apoptosis (Figure 7D). Our results thus revealed that LCL85 targets xIAP and cIAP1 to sensitize metastatic human colon carcinoma cells to Fas-mediated apoptosis.

RT-PCR analysis indicated that LCL85 does not alter the mRNA levels of IAP proteins in human colon carcinoma cells (Figure 7E). Proteasome inhibitor MG-132 blocked LCL85-induced xIAP degradation, whereas caspase inhibitor Z-VAD did not block LCL85-induced xIAP degradation (Figure 7F). Our data thus suggest that LCL85 mediates proteasome-dependent degradation of xIAP protein.

**Figure 8:**
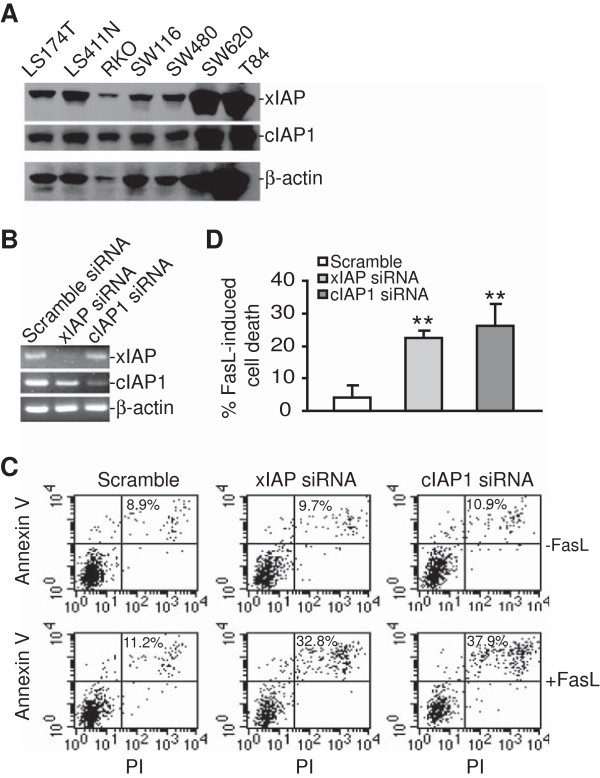
**xIAP and cIAP1 confer metastatic human colon carcinoma cells apoptosis resistance. A**. IAP protein levels in human colon carcinoma cells. The indicated human colon carcinoma cell lines were analyzed for xIAP and cIAP protein levels by Western blotting analysis. β-actin is used as a normalization control. **B**. SW620 cells were transiently transfected with scramble siRNA or xIAP- and cIAP1-specific siRNAs, respectively overnight, and analyzed for xIAP and cIAP1 mRNA levels by RT-PCR. **C**. The transfected cells as shown in A were then treated with FasL (250 ng/ml) for approximately 24 h, stained with Annexin V and PI, and analyzed by flow cytometry. **D**. Quantification of apoptosis. Percentage of FasL-induced cell death as shown in B is calculated by the formula: % Annexin V^+^ and PI^+^ cells in the presence of FasL- % Annexin V^+^ and PI^+^ cells in the absence of FasL. **p < 0.01.

To determine the IAP protein levels in various human colon cancer cell lines, we analyzed xIAP and cIAP1 protein levels in 5 other human colon carcinoma cell lines. Western blotting analysis indicated that xIAP and cIAP1 are expressed in all 5 cell lines at a level similar to that in LS411N and SW620 (Figure 8A).

To validate the functions of xIAP and cIAP1 in Fas-mediated apoptosis in human colon carcinoma cells, SW620 cells were transfected with xIAP- and cIAP1-specific siRNAs, respectively (Figure 8A), and analyzed the tumor cell sensitivity to FasL-induced apoptosis (Figure 8B). Silencing xIAP or cIAP1 significantly increased the tumor cell to FasL-induced apoptosis (Figure 8C). Our data thus suggest that IAP proteins mediate apoptosis resistance in metastatic human colon carcinoma cells, and ceramide sensitizes the tumor cell to Fas-mediated apoptosis at least partially through inducing cIAP1 and xIAP degradation.

### LCL85 also targets Bcl-xL

**Figure 9:**
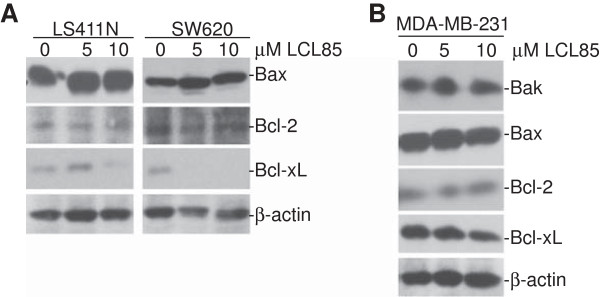
**LCL85 treatment decreases Bcl-xL protein levels in metastatic human colon and mammary carcinoma cells. A & B**. Metastatic human colon **(A)** and mammary **(B)** carcinoma cells were treated with LCL85 at the indicated doses for approximately 24 h and analyzed for the indicated proteins by Western blotting analysis.

Ceramide has been shown to regulate Bcl-x alternative splicing to decrease Bcl-xL level [38], and to mediate Bak and Bax function in the intrinsic apoptosis pathway [39-41]. In addition, Bcl-2 has been shown to activate Bak to induce C16 ceramide accumulation [42,43]. We then analyzed these Bcl-2 family proteins. Western blotting analysis revealed that only Bcl-xL protein level is dramatically decreased by LCL85 in metastatic human colon cancer cells (Figure 9A), and in the metastatic breast cancer cells, albeit to a less degree (Figure 9B).

### Ceramide analog and Smac mimetic additively sensitize metastatic human colon carcinoma cells to apoptosis induction

**Figure 10:**
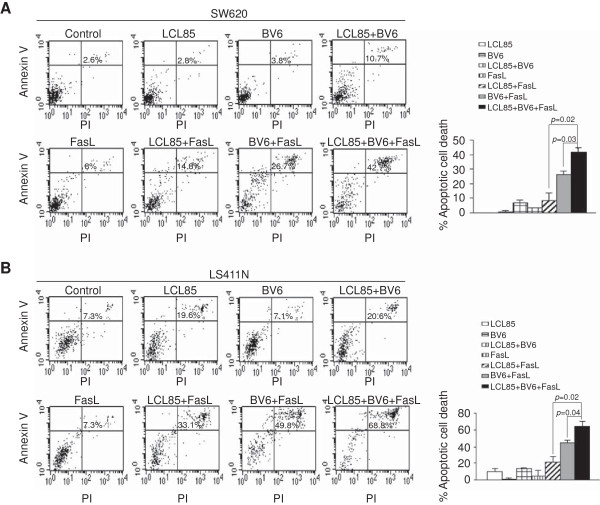
**Ceramide analog and Smac mimetic exhibit additive effects in sensitization of metastatic human colon carcinoma cells to Fas-mediated apoptosis. A & B**. SW620 **(A)** and LS411N **(B)** cells were cultured in the presence of LCL85 (5 μM), BV6 (5 μM), FasL (250 ng/ml), and the combinations as indicated for approximately 24 h. Cells were stained with Annexin V and PI and analyzed by flow cytometry. Apoptotic cell death for each treatment was indicated at the top right corner of each plot. Percentage of FasL-induced cell death is calculated by the formula: % Annexin V^+^ and PI^+^ cells in the presence of FasL- % Annexin V^+^ and PI^+^ cells in the absence of FasL, and presented at the right.

Our observations that LCL85 and BV6 both target IAP proteins suggest that they may act additively in sensitization of tumor cell to apoptosis induction. To test this hypothesis, SW620 and LS411N cells were treated with these two agents alone or in combination, and analyzed for the tumor cell sensitivity to FasL-induced apoptosis. Although sublethal doses of LCL85 and BV6 are both effective in sensitization of tumor cells to FasL-induced apoptosis (Figure 10A & B), clearly, combined LCL85 and BV6 exhibited significantly greater effects than each agent alone on sensitization of these two tumor cells to FasL-induced apoptosis (Figure 10A & B).

### Sensitivity of mouse tumor cells to LCL85-sensitized and Fas-mediated apoptosis

**Figure 11:**
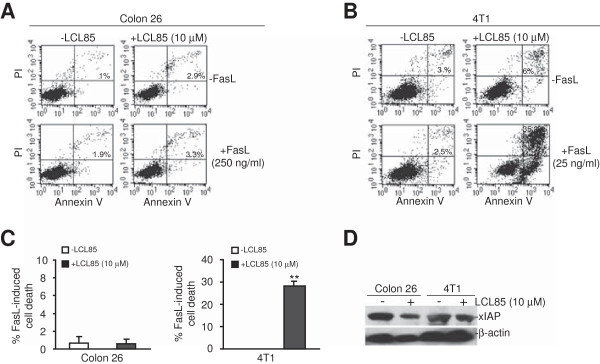
**LCL85 treatment decreases xIAP protein levels in mouse tumor cells. A & B**. Colon 26 **(A)** and 4 T1 **(B)** cells were cultured in the absence or presence of LCL85 (10 μM) and FasL (Colon 26 = 250 ng/ml; 4 T1 = 24 ng/ml) as indicated for 48 h. Cells were then stained with Annexin V and PI and analyzed by flow cytometry. The apoptotic cell death is indicated at the top right corner of each plot. **C**: Quantification of apoptosis. % FasL-induced cell death is calculated by the formula: % Annexin V^+^ and PI^+^ cells in the presence of FasL - % Annexin V^+^ and PI^+^ cells in the absence of FasL. **D**. Colon 26 and 4 T1 cells were cultured in the absence or presence of LCL85 (10 μM) for approximately 24 h and analyzed by Western blotting for xIAP protein level. β-actin was used as a normalization control.

We next sought to test the anti-cancer efficacy of LCL85 in preclinical mouse tumor models. First, we tested whether LCL85 sensitizes mouse tumor cells to FasL-induced apoptosis. Both Colon 26 and 4 T1 cells are resistant to Fas-mediated apoptosis (Figure 11A & B). LCL85 did not exhibit sensitization activity in Colon 26 cells to FasL-induced apoptosis in our initial attempts (Figure 11A). However, A sublethal dose of LCL85 effectively overcame 4 T1 cells resistance to Fas-mediated apoptosis (Figure 11B & C). Western blotting analysis indicated that LCL85 decreased xIAP protein levels in both Colon 26 and 4 T1 cells (Figure 11D).

### Toxicity of LCL85

We analyzed serum enzyme profiles to determine LCL85 liver toxicity. Analysis of serum enzyme/protein levels in mice after LCL85 treatment revealed that LCL85 induces elevated alanine aminotransferase (ALT) in mouse serum in a dose-dependent manner, and an almost 3 fold ALT increase was detected at the highest LCL85 dose examined (5 mg/kg body weight). No other serum enzymes and proteins were significantly elevated by LCL85 (Table 1).

**Table 1:** Mouse serum toxicity profiles of LCL85^*^

	**Treatment**		
**Serum enzyme/protein level**		**LCL85 (mg/kg body weight)**	
	**Control (n = 2)**	**1 (n = 4)**	**5 (n = 3)**
ALP (U/L)	65 ± 7.1	67.5 ± 11.6	66.7 ± 5.1
ALT (U/L)	20.5 ± 4.9	37.8 ± 10.2	57.3 ± 24.2
GGT (U/L)	<3.0	<3.0	<3.0
Albumin (g/dl)	3.2 ± 0.1	3.1 ± 0.1	3.2 ± 0.2
Glucose (mg/dl)	205 ± 29.7	198.5 ± 48.7	176.7 ± 23
Sodium (mmol/L)	150.5 ± 0.7	152.3 ± 2.2	151 ± 3
Potassium (mmol/L)	5.2 ± 0.1	6.4 ± 1	6.9 ± 0.5
Chloride (mmol/L)	108.5 ± 0.7	111 ± 2.6	111.7 ± 2.9
Bicarbonate (mmol/L)	16 ± 0	14.3 ± 2.2	15.7 ± 2.1
Anion Gap (mmol/L)	31 ± 0	33.5 ± 4.2	30.7 ± 2.5
Calcium ((mg/dl)	10.3 ± 0.1	10.2 ± 0.6	9.9 ± 0.3
Phosphorous (mg/dl)	8.8 ± 3.7	9.2 ± 1.9	8.5 ± 0.5
Cholesterol (mg/dl)	74.5 ± 9.2	68.8 ± 12.4	86 ± 4.6
UreaNitrogen (mg/dl)	24.5 ± 3.5	29.3 ± 4	26.3 ± 1.5
Total Bilirubin (mg/dl)	0.1 ± 0	0.1 ± 0	0.1 ± 0
Total Protein (g/dl)	4.5 ± 0.1	4.5 ± 0.3	4.8 ± 0.2

^*^Measurements were performed 3 days after LCL85 injection.

ALP, alkaline phosphatase; ALT, alanine aminotransferase; GGT, gamma-glutamyltransferase.

### LCL85 suppresses colon carcinoma metastatic potential in an experimental lung metastasis mouse model *in vivo*

**Figure 12:**
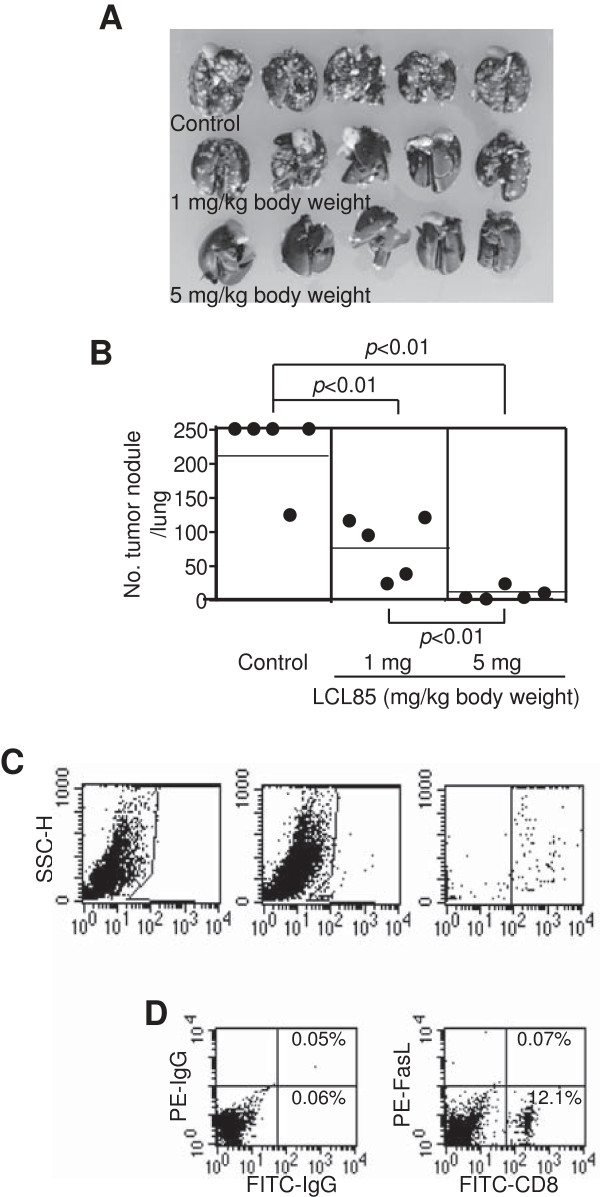
**LCL85 suppresses colon carcinoma cell metastatic potential *in vivo*. A**. Colon26 cells were injected i.v. into mice. Tumor-bearing mice were treated with LCL85 at the indicated doses every 3 days for a total of 4 times. Mice were sacrificed and examined for lung metastases nodules. Shown are tumor-bearing lungs from one of two experiments. **B**. The number of lung tumor nodules are enumerated and shown. Each dot represents total counts from a single mouse. Counts greater than 250 are expressed as >/= 250. The horizontal lines in the plot box represent mean tumor nodule numbers. **C**. Analysis of FasL^+^ cells in tumor-bearing lungs. Single cell suspensions were prepared from lungs of tumor-bearing mice as shown in A, stained with CD8- and FasL-specific mAbs and analyzed by flow cytometry. IgG isotype was used as a negative control (left panel). CD8+ T cells (middle panel), FasL + and CD8+ T cells (right panel) are indicated in each plot. Shown are representative results of 1 from 3 mice. **D**. Analysis of FasL^+^ cells in lungs of tumor-free mice. Single cell suspensions were prepared from lungs of tumor-free mice, stained with CD8- and FasL-specific mAbs and analyzed by flow cytometry. IgG isotype was used as a negative control (left panel). FasL + and CD8+ T cells (right panel) are indicated in each plot. Shown are representative results from 1 of 4 mice.

To determine the efficacy of LCL85 in suppression of metastasis *in vivo*, we used an experimental metastasis mouse model. Colon26 cells, a highly metastatic colon carcinoma cell line, were injected i.v. to mice. Tumor-bearing mice were treated with LCL85 over time. Lung metastasis was then analyzed. LCL85 significantly suppressed colon26 lung metastasis in a dose-dependent manner (Figure 12A & B). Although LCL85 possesses direct anti-tumor cytotoxicity (Figure 1) that might contribute to the observed tumor suppression, it is possible that LCL85 might also sensitize the tumor cells to apoptosis induction by FasL of host immune cells, particularly CD8^+^ CTLs. We then dissected tumor-bearing lungs and made single cell suspension with collagenase. Staining cells with CD8- and FasL-specific mAbs revealed that CD8^+^ T cells in tumor-free mice are essentially FasL^-^. In contrast, approximately 31% of tumor-infiltrating CD8^+^ T cells are FasL^+^ (Figure 12C). CD8^+^ cells in tumor-free mice are all FasL^-^ (Figure 12D). Therefore, LCL85 might sensitize colon carcinoma cells to host FasL^+^ CTL-mediated tumor suppression.

### LCL85 suppresses spontaneous breast cancer metastasis *in vivo*

**Figure 13:**
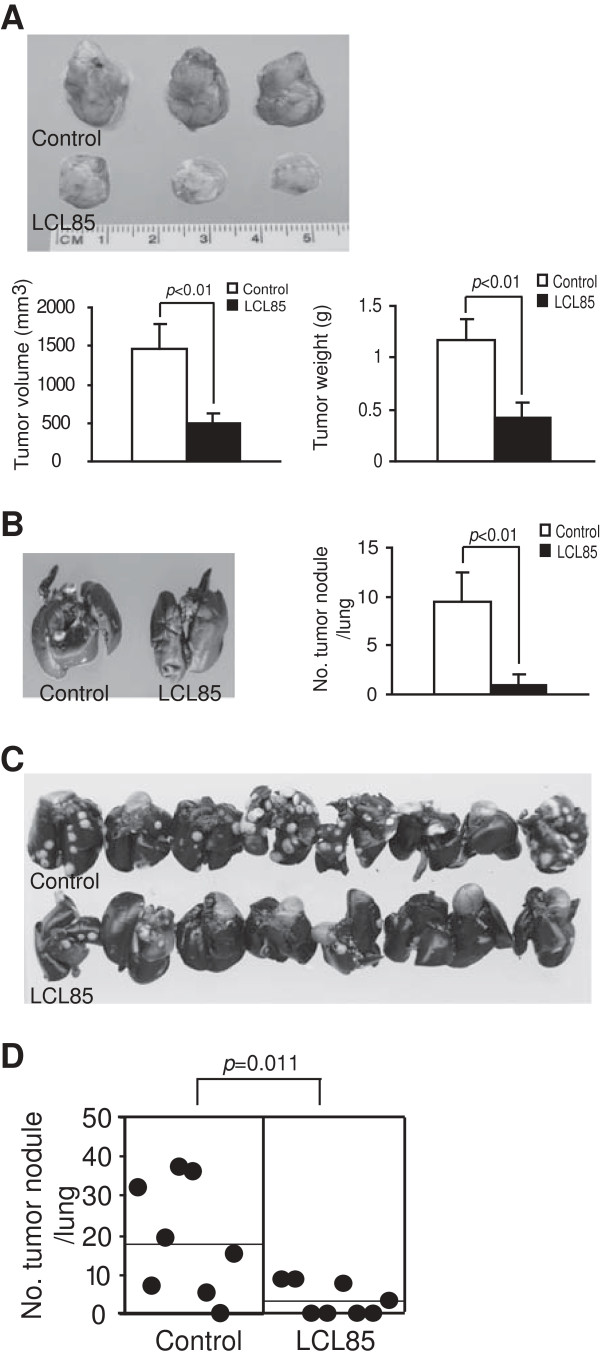
**Ceramide analog suppresses breast cancer growth and spontaneous lung metastasis. A**. LCL85 suppresses breast cancer growth and metastasis. 4 T1 cells were injected to the mammary fat pad of mice. Tumor bearing mice were treated with LCL85 (2.5 mg/kg body weight) through i.v. injection. Tumor sizes were recorded and presented at the bottom left panel. The tumors were also dissected and weighed and presented at the bottom right panel. Column, mean; bar, SD. **B**. Lungs of tumor-bearing mice as in A were analyzed for tumor nodules. The white spots are tumor nodules and the black tissues are normal lung tissues. Shown are images of representative tumor-bearing lungs. The tumor nodules in each lung were enumerated and presented at the right panel. Column, mean; bar, SD. **C**. LCL85 suppresses spontaneous breast cancer metastasis. 4 T1 cells were transplanted to the mammary fat pads of mice. Primary tumors were surgically removed 15 days later. Mice were treated with LCL85 (2.5 mg/kg body weight) at days 8, 11, 14 and 17 after surgery. Lungs were analyzed for metastasis at day 19 after surgery. Shown are tumor-bearing lungs. **D**. The tumor nodules were enumerated. Each dot represents total tumor nodule number of a mouse lung.

To further determine the function of LCL85 in suppression of cancer metastasis, we used a complimentary breast cancer lung metastasis mouse model. Murine breast cancer 4 T1 cells were injected to the mammary fat pad. Tumor-bearing mice were treated with LCL85 over time and both primary tumor growth and lung metastasis were examined. LCL85 significantly suppressed the primary mammary tumor growth *in vivo* as measured by tumor size and tumor weight (Figure 13A). Interestingly, the spontaneous lung metastasis was also significantly suppressed by LCL85 (Figure 13B). The observation that LCL85 suppresses spontaneous breast cancer lung metastasis is significant. However, it is possible that the decreased lung metastasis (Figure 13B) was due to the decreased primary tumor growth (Figure 13A). To determine whether LCL85 directly suppresses spontaneous metastasis, 4 T1 cells were injected to mouse mammary fat pad. Primary tumors were surgically removed 15 days after tumor cell injection. Mice were treated with LCL85 over time after surgery. This procedure thus mimics human breast cancer patient treatment. Analysis of lungs indicated that LCL85 significantly suppresses breast cancer spontaneous lung metastasis (Figure 13C & D). Taken together, our data demonstrated that LCL85 at a subtoxic dose is effective in suppression of colon and breast cancer metastasis.

## Discussion

Ceramide mediates apoptosis through multiple mechanisms. It has been reported that ceramide mediates Fas receptor clustering, capping and activation to promote Fas-mediated apoptosis [21-23]. Ceramide has also been shown to regulate Bcl-x alternative splicing to decrease Bcl-xL level [38], and mediates Bak, Bax and Bcl-2 functions in the intrinsic apoptosis pathway [39-43]. The effects of ceramide on these apoptosis mediators are apparently cell type- or cellular context-dependent since LCL85 only alters the expression level of Bcl-xL in human colon and breast cancer cells. Here, we identified xIAP and cIAP1 as targets of the ceramide signaling pathways in both metastatic human colon and breast cancer cells. We observed that LCL85 effectively decreased cIAP1 and xIAP protein levels in metastatic human colon and breast cancer cells. Consistent with the decreased xIAP1 and cIAP1 protein levels, metastatic human colon carcinoma cells exhibited increased sensitivity to FasL-induced apoptosis. Furthermore, treatment of metastatic human colon carcinoma cells with cIAP1 and xIAP-specific inhibitor BV6 also significantly increased tumor cell sensitivity to FasL-induced apoptosis [44]. Therefore, our data suggest that xIAP1 and cIAP1 proteins are responsible, at least in part, for the apoptosis-resistant phenotype in metastatic human colon and breast cancers, and LCL85 overcomes metastatic human colon and breast cancer cell resistance to Fas-mediated apoptosis at least partially through inducing proteasomal degradation of xIAP and cIAP1 proteins.

It has been well-documented that Smac mimetic BV6 specifically targets cIAP1 and cIAP2 proteins to induce apoptosis through activating the TNFα signaling pathway [36,45]. However, it has also been shown that xIAP, rather than cIAP1 and cIAP2, is the critical target of BV6 in Fas-mediated apoptosis [44,46]. Strikingly, we observed that LCL85 also sensitizes tumor cells to Fas-mediated apoptosis through inducing proteasomal degradation of xIAP. LCL85 treatment increased endogenous C16 ceramide level and exogenous C16 ceramide is effective in sensitizing the apoptotic resistant metastatic human colon carcinoma cells to Fas-mediated apoptosis. Therefore, it is possible that LCL85 sensitizes tumor cells to Fas-mediated apoptosis at least in part through inducing C16 ceramide accumulation, resulting in ceramide-mediated xIAP and cIAP1 proteasomal degradation. However, the molecular mechanisms underlying the crosstalk network between ceramide analog, C16 ceramide and IAP proteins remain to be elucidated.

Ceramide analog-mediated direct cytotoxicity often depends on administering a high dose of the agent [47]. In this study, LCL85 exhibited potent anti-tumor cytotoxicity, suggesting that LCL85 is potentially an effective therapeutic agent in cancer therapy. However, LCL85 also exhibited toxicity in a dose-dependent manner. Therefore, LCL85 might also be toxic if used in high doses. Interestingly, we demonstrated that a sublethal dose of LCL85 is not cytotoxic but effectively sensitizes metastatic human colon carcinoma cells to FasL-induced apoptosis *in vitro*. This observation is significant since a sublethal dose of LCL85 might be safe and yet an effective sensitizer in FasL^+^ CTL-based cancer immunotherapy.

Tumor-reactive CTLs primarily use the perforin and Fas/FasL effector mechanisms to induce target tumor cell apoptosis. Immunosuppression of CTL activation and effector functions by immuno- suppressive cells is a major challenge in cancer immunotherapy. However, recent studies revealed that the immuno- suppressive Treg cells only selectively suppress the perforin pathway without inhibiting CTL activation and proliferation *in vivo*[48,49], suggesting that Treg cells may not suppress the Fas/FasL effector mechanism of CTL *in vivo*. Indeed, our recent study showed that tumor-infiltrating CTLs in tumor-bearing mice and CTLs from human colon and breast cancer patients are FasL^+^[32]. Therefore, the Fas/FasL effector mechanism might be functional in the immuno suppressive tumor microenvironment. However, metastatic human colon and breast cancer cells are often resistant to Fas-mediated apoptosis [8,9]. Therefore, a therapeutic agent that can sensitize tumor cell Fas resistance may represent an effective enhancer of CTL-based cancer immunotherapy against metastatic colon and breast cancers. Our data suggest that LCL85 is potentially such an agent. Although LCL85 does not effectively sensitize Colon 26 cells to FasL-induced apoptosis, LCL85 is effective in suppressing Colon 26 cell metastatic potential in vivo, suggesting that other host factors, such as IFN-γ and TNFα secreted by T cells, might also act to sensitize the tumor cells to apoptosis in vivo, which requires further study.

## Conclusions

We envision that a sublethal dose of LCL85 can be used as a sensitizer in cancer immunotherapy for metastatic colon and breast cancers. This idea is analogous to a “one-two punch” concept. First, cancer patients are treated with a non-cytotoxic dose of LCL85 to sensitize cancer cells to apoptosis. Once “sensitized”, patients are then treated with FasL^+^ CTLs-based immunotherapy to suppress cancer metastasis. Our *in vivo* tumor suppression studies showed that low doses (2.5-5 mg/kg body weight) of LCL85 exhibited potent tumor suppression activity in immune-competent mice *in vivo* (Figure 12). A previous study showed that lack of ceramide accumulation in target cells is a significant cause of resistance to cytotoxic T lymphocyte (CTL)-induced apoptosis [50]. In this study, we observed that a large portion of the tumor-infiltrating CTLs are FasL^+^ (Figure 12C), and low doses of LCL85 effectively suppresses colon and breast tumor growth and metastasis in immune-competent mice (Figures 12 & 13). Our observations thus indicate that LCL85 might sensitize tumor cells to CTL-induced apoptosis through inducing ceramide accumulation in the tumor cells *in vivo,* which requires further investigation. Nevertheless, our data suggest that LCL85, although effective as a single agent in suppression of tumor development at high doses, might be more valuable if used at a sublethal dose as a sensitizer for enhancing the efficacy of FasL-based cancer therapy, particularly CTL-based cancer immunotherapy.

## Competing interests

The authors declare that they have no competing interests.

## Authors’ contributions

Concept and design: EB, ABi, and KL. Development of methodology: AVP, MAZ, DY, CMT, MRC, EB, ABa, JB, and KL. Acquisition of data: AVP, MAZ, DY, CMT, JB, ABa, XL and KL. Analysis and interpretation of data: AVP, MAZ, CMT, EB, ABi, and KL. Writing and review of manuscript: MAZ, EB, ABi, and KL. All authors read and approved the final manuscript.

## Pre-publication history

The pre-publication history for this paper can be accessed here:

http://www.biomedcentral.com/1471-2407/14/24/prepub
